# Current Strategies for the Gene Therapy of Autosomal Recessive Congenital Ichthyosis and Other Types of Inherited Ichthyosis

**DOI:** 10.3390/ijms23052506

**Published:** 2022-02-24

**Authors:** Daria S. Chulpanova, Alisa A. Shaimardanova, Aleksei S. Ponomarev, Somaia Elsheikh, Albert A. Rizvanov, Valeriya V. Solovyeva

**Affiliations:** 1 Institute of Fundamental Medicine and Biology, Kazan Federal University, 420008 Kazan, Russia; daryachulpanova@gmail.com (D.S.C.); aliceshaimardanova@mail.ru (A.A.S.); l.ponomarev2013@gmail.com (A.S.P.); rizvanov@gmail.com (A.A.R.); 2 Division of Cancer and Stem Cell, University of Nottingham, Nottingham LE12 5RD, UK; somaia.elsheikh@nottingham.ac.uk

**Keywords:** autosomal recessive congenital ichthyosis, lamellar ichthyosis, transglutaminase-1, gene therapy, cell therapy

## Abstract

Mutations in genes such as transglutaminase-1 (*TGM1*), which are responsible for the formation and normal functioning of a lipid barrier, lead to the development of autosomal recessive congenital ichthyosis (ARCI). ARCIs are characterized by varying degrees of hyperkeratosis and the presence of scales on the body surface since birth. The quality of life of patients is often significantly affected, and in order to alleviate the manifestations of the disease, symptomatic therapy with moisturizers, keratolytics, retinoids and other cosmetic substances is often used to improve the condition of the patients’ skin. Graft transplantation is commonly used to correct defects of the eye. However, these approaches offer symptomatic treatment that does not restore the lost protein function or provide a long-term skin barrier. Gene and cell therapies are evolving as promising therapy for ARCIs that can correct the functional activity of altered proteins. However, these approaches are still at an early stage of development. This review discusses current studies of gene and cell therapy approaches for various types of ichthyosis and their further prospects for patient treatment.

## 1. Introduction

Ichthyosis is a heterogeneous group of diseases, both hereditary and acquired, characterized by dry, rough skin with noticeable scaling, covering large parts of the body [1]. The development of acquired ichthyosis is associated with malignant neoplasms, autoimmune, metabolic, endocrine and infectious diseases [2]. The heterogeneous manifestation of ichthyosis leads to difficulties in diagnosis and confusion in terminology in different countries. In this review, we are using the classification accepted at First Ichthyosis Consensus Conference in Sorèze in 2009 [3], with the addition of information on mutations associated with the development of ichthyosis described since 2010. An umbrella term autosomal recessive congenital ichthyosis (ARCI) is used to describe a heterogenous group of ichthyoses induced by germline mutations in at least ten genes. Mutations in arachidonate 12-lipoxygenase, 12R type (*ALOX12B*), arachidonate lipoxygenase 3 (*ALOXE3*), NIPA-like domain containing 4 (*NIPAL4*)/*ichthyin*, patatin like phospholipase domain containing 1 (*PNPLA1*) and cytochrome P450 family 4 subfamily F member 22 (*CYP4F22*) genes are associated with the development of non-bullous congenital ichthyosiform erythroderma (NBCIE; OMIM #242100, OMIM #606545, OMIM #612281, OMIM #615024, OMIM #604777) [4,5]. Truncating mutations in the ATP-binding cassette subfamily A member 12 (*ABCA12*) gene tend to cause Harlequin ichthyosis (HI, OMIM #242500), while *ABCA12* missense mutations are associated with lamellar ichthyosis (LI; OMIM #601277) [6,7]. Mutations in *NIPAL4/ichthyin* gene also lead to the development of ARCI type III [8]. Mutations in transglutaminase-1 (*TGM1*) gene lead to the development of LI and rarer ARCI form bathing suit ichthyosis (OMIM #242300) [9]. In addition to these main forms of ARCI, several rare subtypes have been identified, such as self-improving collodion ichthyosis (*ALOX12B*, *ALOXE3*, and *TGM1* mutations) [10].

Despite the variety of mutations and phenotypic manifestations of ARCI, from 32% to 68% of cases (~70–90% of LI) are caused by mutations in the *TGM1* gene [11]. In the United States, *TGM1* mutations are responsible for 55% of ARCI cases [12,13]. To date, more than 115 mutations in the *TGM1* gene have been described in patients from diverse racial and ethnic backgrounds (Caucasion Americans, Norwegians, Swedish, Finnish, German, Swiss, French, Italian, Dutch, Portuguese, Hispanics, Iranian, Tunisian, Moroccan, Egyptian, Afghani, Hungarian, African-Americans, Korean, Japanese and South African) [14]. However, the highest LI incidence rate is found in Norway (1:91,000) [15] and Galicia (northern Spain) (1:122,000) [12], with both cases being due to the founder effect. Presumably, the mutation in Galicia could have arisen about 1000 years ago and then spread to the Ecuadorian province of Manabí, where LI is also highly prevalent [16].

**Figure 1:**
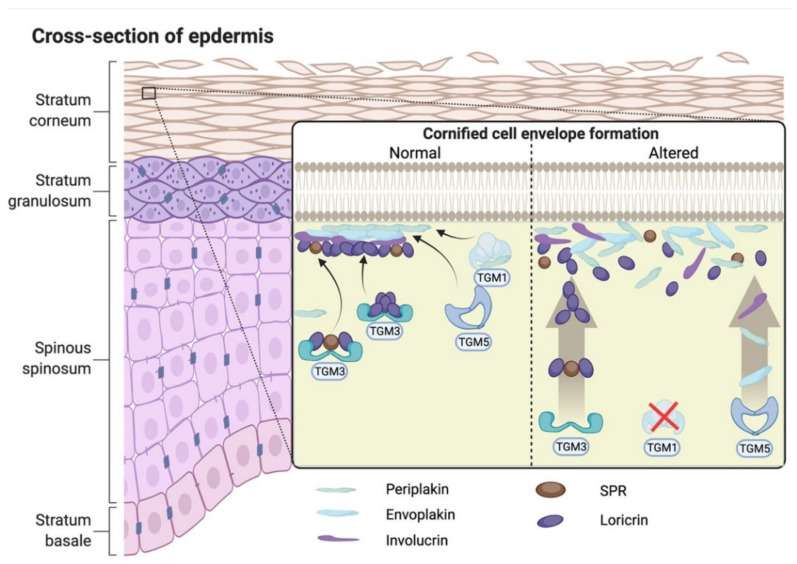
The formation of cornified cell envelope (CCE), a physical and water-impermeable structure, is important as a scaffold for the following formation of a normal lipid layer in the stratum corneum of the skin. Normal transglutaminase-1 enzyme catalyzes Nε-(γ-glutamyl) lysine crosslinking precursor proteins, such as loricrin and involucrin. These linked peptides, in turn, attach to the outer side of the CCE. Mutation in *TGM1* gene lead to altered transglutaminase-1 enzyme function, formation of defective intercellular lipid layers and disruption of the stratum corneum barrier function. TGM1–transglutaminase-1, TGM-3–transglutaminase-3, TGM5–transglutaminase-5.

The *TGM1* gene is located on chromosome 14q11.2 (GenBank NM_000359.3) and encodes transglutaminase-1 (TGase-1) enzyme, which has a molecular weight of ~90 kDa (GenBank NM_000359.3). TGase-1 is Ca^2+^-dependent enzyme, which is expressed at epidermal keratinocytes in the skin, and stratified squamous epithelium of the upper digestive tract and in lower female genital tract [17]. TGase-1 catalyzes Nε-(γ-glutamyl) lysine crosslinking precursor proteins, such as loricrin and involucrin. These linked peptides form the cornified cell envelope (CCE), a physical and water-impermeable structure that is important for the skin barrier function. The CCE formation is important as a scaffold for the following attachment of lipid molecules to form a normal cornified lipid envelope (CLE) in the stratum corneum of the skin [14]. Altered TGase-1 enzyme function leads to the formation of defective intercellular lipid layers and disruption of the stratum corneum barrier function (Figure 1) [18]. Severe TGase-1 disfunction can lead to the classic LI phenotype [19]; partial loss of TGase-1 activity can result in the development of mild LI [20] or NBCIE phenotype [21].

After mutations in the *TGM1* gene, the most common causes of ARCI are mutations in *ALOXE3* and *ALOX12B* (17–30%), *NIPAL4*/*ichthyin* (10–16%), *CYP4F22* (8–10%)*, ABCA12* (5%) genes. The number of cases where a mutation is unknown varies from 15 to 22% [22,23]. Gene mutations associated with ARCI and linked ARCI phenotypes are summarized in Table 1.

This review aims to describe current ARCI conventional therapy, gene and cell therapy approaches for various types of ichthyosis and discuss their further prospects for the patient treatment.

## 2. Pathophysiology of the Diseases and Diagnosis

Harlequin ichthyosis has the most severe phenotypic manifestations among ARCIs. Newborns with HI always have severe collodion membrane and extreme ectropion and eclabium. Large gray or yellowish scales are disturbed throughout the body. Sever erythema is also observed. Due to severe skin lesions, temperature regulation is impaired, and being prone to skin infections is also observed. Another complication that occurs in both HI patients and other types of ARCI is anhidrosis, the inability to sweat [11]. The development of anhidrosis is probably associated with an invisible hyperkeratotic plugging and capping of the sweat duct [32]. The mortality rate of HI newborns is about 50%, which is the highest compared to other ARCI types [33]. HI diagnosis is usually not difficult because of its phenotype. However, prenatal detection of *ABCA12* mutations is a crucial diagnostic criterion. Prior to the identification of *ABCA12* as a gene causing the disease, prenatal HI diagnosis was performed by electron microscopy of fetal skin biopsy samples [34]. The ultrastructure of lamellar bodies in HI cells is impaired due to the deficiency of intercellular lamellae in the stratum corneum [35]. Three-dimensional sonography construction can also be used to diagnose severe ARCI, including HI, by the presence of dense floating particles in amniotic fluid (“snowflake sign”), which indicates abnormal corneocyte shedding (disadhesion) [36].

Newborns with a mutation in the *TGM1* gene often have ectropion and collodion membrane [37]. Subsequently, the membrane dries and exfoliates, and is then replaced by brown lamellar scales. Scales can be limited to trunk, legs, forearms, and forehead in mild LI or involve the whole body in severe forms [38]. Ectropion, eclabium, cicatricial alopecia of the scalp and eyebrows, and palmar and plantar hyperkeratosis, can occur in severely affected children. The nails may be curved, and the ears are often wrinkled and close to the scalp. Mild erythroderma may be present [39]. LI diagnosis is also based on fetal skin biopsy and electron microscopy observation during the later stages of pregnancy. Ultrastructural analysis of cells from ARCI patients showed that CCE is absent in patients with LI, and keratinocytes have a reduced level of TGase-1 enzyme activity [40]. Absence of or significant defects in CCE are also observed in epidermal scales and nails of patients with a mutation in the *TGM1* gene [41]. Moreover, cholesterol clefts, lipid vacuoles in cornified cells, malformed cornified cell envelope and abnormal lamellar granules were detected in skin cells of LI patients [42]. Depending on the type of mutation, keratinocytes of ARCI patients showed a 0.5–4% reduction in the membrane enzymatic activity of TGase-1 compared to healthy controls [14]. Histological analysis of the skin of LI patients has shown hyperkeratosis, focal parakeratosis, a normal or thickened granular layer with increased mitoses, acanthosis, psoriasiform hyperplasia and perivascular lymphocytic infiltrate [43].

Children with NBCIE are frequently born as collodion babies. After the collodion membrane is dropped, erythroderma and scaling appear. Scales are typically fine and white or light gray and can be localized or generalized [3]. NBCIE diagnosis is also based on the analysis of cell ultrastructure, which shows mild to moderate hyperkeratosis, a normal or moderately thickened granular cell layer, slight acanthosis and variable parakeratosis [44].

Due to the absence of robust genotype–phenotype correlation and the genetic heterogeneity of ARCI, accurate diagnosis requires identification of the gene, the mutation in which led to the development of the disease [23]. The recent development of prenatal diagnostic methods allows a DNA analysis to be performed for most common ARCI diseases using chorionic villus sampling and amniocentesis in early pregnancy, with a reduced risk for both mother and child. Today, the usual step in diagnosis process is the analysis of a multigene panel covering the known genes associated with the development of ichthyosis [45]. If it is not possible to identify already known mutations, then, using modern methods of DNA analysis, such as next-generation sequencing (NGS), partial sequencing of the exome can be performed [46]. To date, due to the extensive screening for mutations associated with ichthyosis, disease-causing mutations can be found in 90% of ARCI patients [32].

## 3. Potential Biomarkers of the Diseases

In addition to obvious morphological signs, an alteration in the barrier function of the skin leads to the dysregulation of cytokines and/or immune cells and changes in lipid metabolism in the affected cells. Recent studies have revealed changes in the expression of cytokine genes and the number of immune cells that can be used as biomarkers to diagnose and monitor ARCI course. For example, increased CD3^+^ T-cell and dendritic cell infiltration and the increased expression of genes encoding common inflammatory markers as interleukin *(IL)-2* and *IL-15* and innate immunity cytokines *IL-1β* and *IL-8* have been found in ARCI patients [47]. In addition, an upregulation of T-helper (Th) type 17 pathway genes (*IL17F* and *IL36B*/*G*) and IL-17-related proteins synergistically induced by IL-17 and tumor necrosis factor (TNF)-α (IL-17A/C, IL-19, CXC motif chemokine ligand 1 (CXCL1), peptidase inhibitor 3 (PI3), chemokine (CC motif) ligand 20 (CCL20)) in the cells of patients with different types of ARCI, which correlates with the severity of the disease, was observed [48].

Alterations in the expression of genes that regulate lipid metabolism (elongation of very long chain fatty acids elongase 3 and galanin genes) were found with a parallel decrease in extracellular lipid level and keratinocyte compaction [48]. In patient with *NIPAL4*/*ichthyin* gene mutation, stratum corneum amounts of ceramides with carbon chain-length (C) 32–52 were increased, while amounts of most acylceramide with C66:2–C72:2 were reduced [49].

## 4. Animal Model of ARCI

To improve existing therapy and produce fundamentally new approaches for the treatment of congenital ichthyosis, a high-quality, stable preclinical model that can fully reproduce the phenotype of human impaired skin is required.

To determine the specific mechanisms leading to the development of the LI phenotype in keratinocytes, a rat epidermal keratinocyte model was created by suppressing the expression of the *Tgm1* gene. The resulting model reproduced skin areas that were similar to human epidermis. The *Tgm1* gene knockdown by siRNA led to a change in the expression of the components of the stratum corneum, hyperkeratosis, and an increase in the synthesis of neutral lipids in organotypic culture. An analysis of such keratinocytes revealed an upregulation of *IL-1α* expression, and an increase in *IL-1α* expression in all patients with ARCIs including LI. It is interesting that the treatment of keratinocytes with an IL-1α receptor antagonist prevented hyperkeratosis without affecting the levels of non-polar lipid synthesis, which can allow for the creation of new therapies for LI [50].

*Tgm1*^−/−^ mice have similar skin features to severe forms of ichthyosis but die within the first hours of life [51] due to impaired skin barrier function. Studies using these models made it possible to establish pathological features of the skin, such as the absence of corneocyte lipid envelope and cornified envelope, significant hyperkeratosis and hyperplasia of the epidermis. In addition, TGase-1 has been found to play an important role in the formation of a functional skin barrier. However, studies in such animals can only be performed neonatally [52,53]. Thus, these models can be used to find important markers of the disease, which can lead to the creation of new therapeutic strategies, but cannot be used to evaluate treatment effectiveness.

The transplantation of the skin of patients with ichthyosis to laboratory animals can be an alternative model. Using tissue engineering and surgical tools, models with stable human skin engraftment in immunodeficient nu/nu mice have been obtained [54]. Aufenvenne et al. also obtained a humanized mouse model to study the molecular mechanisms of *TGM1*-deficient LI [55]. Human LI skin grafts were maintained in athymic nude mice from 6 weeks to 4 months with excellent retention of all common morphological and histological signs of the disease. Recombinant grafts consisting of an LI patient’s and normal skin cells were grown for periods up to 87 days in nude mice; the abnormal epidermis retained all LI features [56].

A possible cross-transfer of the *TGM1* gene into the intact LI patient epidermis was also investigated. Choate et al. used keratinocytes from *TGM1*-deficient LI patients along with normal keratinocytes to regenerate human LI epidermis in a nude mouse model [57].

In general, the mechanisms involved in the development of human skin diseases and related diseases in mice are very similar [58]. In addition, the phenotype of human skin cells induced by the ichthyosis gene knockdown in vitro is very similar to that described in mice with knockdown of the same gene. However, there are differences in the structure of the skin of mice and humans, which include a reduced water barrier or higher percutaneous absorption in mice, which creates limitations in the evaluation of local drug delivery [59]. Therefore, the models of LI patient skin grafts seem to be more appropriate for studying the effectiveness of the drugs being developed for the treatment of LI and ARCI.

## 5. Conventional Therapy

### 5.1. Symptomatic Therapy

Current ARCI therapy is symptomatic and does not provide a long-term effect. It aims to improve patient’s quality of life without treating the underlying cause. Current treatment includes moisturizers, keratolytics, retinoids, vitamin D analogs, corticosteroids, and calcineurin inhibitors [60].

Retinoids allow for improvements in the condition of the patients’ skin due to their ability to reduce the adhesion of horny cells and, therefore, to increase their exfoliation, inhibit the proliferation of the epithelium, help normalize terminal differentiation of skin cells. In addition, retinoids are able to activate the production of epidermal enzymes, which is impaired due to *TGM1* mutation. The molecular mechanisms of retinol action are directly related to all-trans retinoic acid (atRA) and 3,4-didehydroretinoic acid (ddRA) [61]. In cells (primarily in differentiated keratinocytes), retinol is metabolized to atRA and ddRA [62]. Further, atRA and ddRA bind to nuclear retinoic acid receptors (RARα, -β and -γ) and retinoid X receptors (RXRα, -β and -γ) and activate them. Most often, these receptors are assembled into RARγ/RXRα heterodimers and are expressed on the cells of the differentiated layers of the normal epidermis [63,64]. Activation of RAR, RXR receptors regulates the process of terminal differentiation of keratinocytes. In addition, retinoids interact with proteins of various signaling pathways, for example, with activator protein-1 (AP-1) [65]. AP-1 regulates the expression of genes that induce keratinocyte differentiation, such as TGase-1, loricrin, keratin 1, and involucrin [66], all of which are suppressed by atRA [67,68,69].

Several studies have proven the effectiveness of oral retinoids for ARCI therapy. However, this effect only lasts as long as treatment is continued. In a clinical trial evaluating the effect of acitretin therapy, some LI patients responded to treatment with gradually increasing doses of oral retinoids (≥35 mg/day), others at low doses (10–25 mg/day) [70]. Oral retinoid therapy (isotretinoin, etretinate, or acitretin) at doses ranging from 0.5 to 2.5 mg/kg/day in children with HI resulted in an increase in survival rate by 59% compared to untreated children. At the same time, it should be noted that 63% of deaths of newborns without therapy occurred within three days after birth [71].

However, retinoid therapy has side effects related to an increased inflammatory process in the skin, which leads to itching and has a negative effect on the epithelium [71]; therefore, their use is limited. However, retinoic acid metabolism-blocking agents (RAMBA) open up new possibilities [72]. One such drug is liarazole, which inhibits 4-hydroxylase via cytochrome P450 (CYP) 26, thus preventing retinoic acid catabolism, and exhibits retinoid-sparing effects in vivo [73]. In the presence of low doses of retinoic acid or all-trans retinol, liarozole can amplify the effectiveness of low doses of retinoids, in a manner characteristic of the retinoid at a higher dose [74]. The mRNA expression of the cellular protein-binding retinoic acid II (CRABPII), keratins (KRT) 2 and 4, CYP26A1 and B1, and two markers of inflammation (IL-1α and TNF-α) was compared in 11 patients with LI and 12 healthy volunteers of appropriate age and gender. There was no significant difference between the groups, except for increased *CRABPII* expression. Biomarkers before and after 4 weeks of liarozole treatment, which gave a better therapeutic response in patients with a mutation in the *NIPAL4*/*ichthyin* gene than in patients with *TGM1* gene mutations, were suggested. A significant decrease in the expression of mRNA of the *KRT2* and *TNF-α* genes and a tendency to an increase in the expression of mRNA of the *KRT4* and *CYP26A1* genes were observed in patients receiving liarozole, which is consistent with increased retinoid-mediated stimulation of the epidermis. However, there were no dose-dependent responses, and the results of the analysis on the protein level did not always coincide with the data obtained at the mRNA level. The results of the study indicate that liarozole has a therapeutic effect in LI, mildly affecting the expression of retinoid-regulated genes in the epidermis [75].

Skin inflammation is an important part of the ARCI pathogenesis. Diseases with similar pathogenesis may become a source of new targets for anti-inflammatory therapy. Comparison of biopsies from patients with ichthyosis and patients with psoriasis and atopic dermatitis revealed an IL-17 dominant immune profile in ichthyosis that was very similar to that in psoriasis [47]. Inflammation in psoriasis is caused by pro-inflammatory cytokines such as IL-23, IL-17 and TNF-α, as well as Th17 lymphocytes [76]. Therefore, the modulation of the inflammation can be achieved by suppressing these cytokines. For example, the efficacy of anti-IL-17 therapy has been demonstrated in psoriatic erythroderma [77]. Thus, the search for new therapeutic targets can support the development of cytokine-targeted treatment for ichthyosis. It has been shown in pediatric patient with *ABCA12*-deficiency-related erythrodermic ichthyosis that, after 6 months of treatment with Secukinumab (150 mg weekly), which specifically targets IL-17, there was a 48% decrease in the Ichthyosis Area Severity Index score. Cytokine analysis revealed a decrease in pro-inflammatory cytokines derived from keratinocytes and in the number of Th17 during therapy [78]. The safety of Secukinumab in patients with ichthyosis was also demonstrated in the clinical trial (NCT03041038).

Glycerin, urea and propylene glycol are the most commonly used moisturizing ingredients and are commonly found in cosmetics for the symptomatic treatment of ichthyosis [79,80]. In case of insufficiently effective monotherapy or a particularly strong keratinization of the skin, more specific local exfoliating agents are used. One of these compounds is N-acetylcysteine, which, together with urea, has shown good results in the treatment of children with lamellar ichthyosis [81]. Topical anti-inflammatory drugs (steroids, calcineurin inhibitors) are often ineffective and build up a tolerance [82,83]. Current conventional treatment approaches are described in Table 2.

### 5.2. Graft Transplantation

The most common ocular abnormality in congenital ichthyosis is cicatricial ectropion. Progressive scarring and abnormal keratinization of the eyelid skin result in the progressive ectropion of both eyelids, which subsequently leads to lagophthalmos and corneal exposure. Currently, ARCI therapy using graft transplantation onto the affected areas is being actively developed.

An example of the successful use of autologous transplantation is described by Uthoff et al. in the cicatricial ectropion treatment. Among all the types of ichthyosis, only LI or ichthyosis congenita is associated with the development of ectropion and subsequent pathologies of the organs of the visual system. Surgical correction of ectropion has been performed with grafts taken from the hand, eyelids, postauricular skin and groin. Eye symptoms have been successfully treated [87]. Mother’s skin area was also used for allotransplantation without preliminary HLA analysis or immunosuppressive agent administration. After 10 months, the graft retention was good and the ectropion was corrected [88].

Another case of corneal grafting in a 47-year-old man with ARCI with primary keratouveitis of the right eye with keratolysis and exudation in the anterior chamber was also described. The patient was diagnosed with cicatricial ectropion and significant lagophthalmos. The patient underwent tectonic penetrating keratoplasty, which is used to restore the structural integrity of the cornea, cataract extraction and intraocular lens insertion without postoperative complications [89].

An important feature of skin graft transplantation in the treatment of ectropion is the skin graft exfoliation, since ichthyosis is a keratinization disease and, therefore, the graft also requires frequent use of emollients. Secondary skin graft contracture may also occur. Subramanian et al. recommended applying silicone gel sheets for at least 3 months to reduce postoperative contracture, as well as moisturizers (such as oils or liquid paraffin) [90].

### 5.3. Enzyme Replacement Therapy

Enzyme replacement therapy (ERT) can be an effective method for treating various types of ARCI, since the pathological manifestation of diseases is due to the partial or complete absence of a protein that is necessary for normal keratinization of the epidermis. ERT has been used with relative efficacy to treat patients with various lysosomal storage diseases (LSD) such as Fabry disease (OMIM #301500), type II mucopolysaccharidosis (OMIM #309900) and Gaucher disease (OMIM #230800), Tay-Sachs disease (OMIM #272800), metachromatic leukodystrophy (OMIM #607574) [91,92,93,94,95].

Local ERT of LI can restore TGase-1 activity and correct the structure of TGase-1-deficient skin grafts in animal models [84]. Aufenvenne et al. demonstrated an effective technique for the delivery of a recombinant protein using liposomes to restore TGase-1 activity and regenerate the skin in LI model animals. Liposomes loaded with TGase-1 effectively restored TGase-1 activity in the upper stratified layers of the epidermis, increased the integrity of the epidermis, and improved its barrier function. At the same time, the distribution pattern of a number of epidermal differentiation markers, such as loricrin, filaggrin, involucrin, and plasminogen activator inhibitor 2 (PAI-2), have been normalized [84].

Plank et al. have investigated full-thickness skin equivalents obtained from fibroblasts and keratinocytes from ARCI patients with mutation in *TGM1* [85]. The resulting skin equivalents were treated with the TGase-1 enzyme. Protein delivery was performed using thermosensitive nanogels (tNGs) [96]. After the treatment with tNGs loaded with TGase-1, a significant 50-fold decrease in the permeability of skin equivalents was observed, indicating an improvement in barrier function. This is supported by earlier work using topical tNGs to repair barrier defects in *TGM1* knockdown skin equivalents [97].

## 6. Gene Therapy

To date, there are few studies investigating the effect of gene therapy in ichthyosis. This is due to the fact that hereditary ichthyosis is an extremely rare disease, which is why this disorder is under-explored. The genetic mechanisms leading to the development of various types of ichthyosis are being actively studied, but are still not completely clear. This makes it difficult to develop effective ARCI gene therapy strategies. Nevertheless, gene therapy is a promising ARCI therapy. Firstly, skin treatment has undoubted advantages due to the availability of various manipulations, in comparison with other, more inaccessible tissues and organs. Secondly, ichthyosis is often a monogenic disease caused by mutations in one gene, which makes it possible to compensate the lack of the functional protein to achieve a therapeutic effect. In this section, the existing viral and non-viral approaches to the gene therapy for ichthyosis caused by mutations in various genes will be discussed. First, we focus our attention on ARCI; gene therapy for other ichthyosis is discussed in a separate section.

### 6.1. Retroviruses

Retroviruses can effectively infect epidermal progenitor cells and provide a stable expression of therapeutic genes in the skin for several cycles of skin renewal; therefore, they can be considered a tool for gene delivery for skin diseases [98].

Keratinocytes of ichthyosis patients can be successfully modified using retroviruses. It was shown that, after transduction with retroviruses encoding the *TGM1* gene, the expression and activity of TGase-1 was restored in the mutant keratinocytes of LI patient in vitro [99]. Ichthyosis gene therapy is described in Table 3.

### 6.2. Herpesviruses

A vector based on herpes simplex virus type 1 (HSV-1) encoding the TGM1 gene has been proposed as a safe and effective method for gene therapy of ichthyosis caused by the TGM1 gene mutation. HSV-1 is an attractive vector for the treatment of ichthyosis because it has epitheliotropism and can penetrate skin keratinocytes more efficiently than other viral vectors. The HSV-1 vector is safe as its genetic information does not integrate into the DNA of a host cell, which means the virus does not cause insertional mutagenesis. Preclinical studies have shown that the vector effectively infects TGM1-deficient human skin cells, restores the TGase-1 enzyme activity in vitro, and also significantly increases the expression of TGase-1 without toxicity when administered locally into immunocompetent BALB/c mice in vivo. It has also been described that the vector did not show toxicity and immunogenicity, even when injected weekly into the epidermis of animals, and does not spread to other areas from the injection site [100]. A phase I/II clinical trial has been recently registered (August 2019) to evaluate the safety of topical administration of this genetic vector. To date, this is the only registered clinical trial of the efficacy of direct gene therapy for the treatment of ichthyosis (NCT04047732).

### 6.3. Non-Viral Methods

Non-viral gene transfer methods have a number of advantages, including low toxicity, low immunogenicity, safety and easier manufacturing, compared to viral vectors. The disadvantages of this approaches are the short duration of gene expression and low transfection efficiency [108].

The non-viral system for the *TGM1* gene delivery to the keratinocytes of LI patients was produced and named the adenovirus-enhanced, transferrin-receptor-mediated transfection (AVET) system. AVET is a triple complex consisting of a biotinylated, chemically inactivated adenovirus, non-covalently linked to plasmid DNA encoding the *TGM1* gene and polylysine-transferrin. The in vitro transfection efficiency of keratinocytes using AVET was approximately 28%, which was much better than the results of transfection mediated by other polycationic transfection reagents (SuperFect and PrimeFector). It was also found that the AVET system better allowed for the transfection differentiated cells than stem cells. In organotypic cultures (3D culture), AVET transfection did not show the expected efficiency, which could be due to the presence of a stratum corneum or the absence of a receptor for adenovirus entry [101].

Non-viral method was also used for the *ABCA12* gene delivery to the keratinocytes of patients with HI. It is known that mutations in the *ABCA12* gene result in defective lipid transport, which leads to the development of HI or LI. After genetic modification with a plasmid vector containing the wild-type *ABCA12* gene, the keratinocytes of the HI patient began to express the normal ABCA12 protein, which led to the restoration of lipid secretion from lamellar granules and cell phenotype improvement. This work provided evidence that ABCA12 works as a lipid transporter for epidermal keratinocytes and that defective ABCA12 leads to a loss of the skin lipid barrier [6].

### 6.4. Gene Therapy for Other Types of Ichthyoses

Since there are very few studies on the effectiveness of gene therapy for ARCIs, we decided to include a description of gene therapy approaches for other types of ichthyosis in this review, since the same strategies can be modified for ARCIs.

Adeno-associated viruses (AAV) are the most attractive vectors for gene therapy today. They provide stable expression without insertion of the transgene into the cell genome, have low immunogenicity, and can transduce both dividing and non-dividing cells, including keratinocytes [104,105,109].

AAV serotype 2 (AAV2) was used to deliver the aldehyde dehydrogenase 3 family member A2 (*ALDH3A2)* gene to the keratinocytes of patients with Sjögren–Larsson syndrome. This type of ichthyosis is characterized by mutations in the *ALDH3A2* gene encoding the microsomal nicotinamide adenine dinucleotide (NAD)-dependent enzyme fatty aldehyde dehydrogenase (FALDH), which is required for the oxidation of long-chain aliphatic aldehydes into fatty acids. The disease leads to the development of congenital ichthyosis, intellectual disability and spasticity. The use of recombinant AAV2 containing cDNA encoding the gene for the missing FALDH enzyme resulted in the restoration of the enzyme function in mutant cells. In genetically modified cells, resistance to aldehydes, which are toxic to mutant cells, has been increased almost to the level of normal cells [104,105].

A retrovirus-based vector has been used to develop a therapy for X-linked ichthyosis (XLI), which is characterized by loss of steroid arylsulfatase C function due to mutations in the STS gene. Keratinocytes of XLI patients were modified using retrovirus encoding the STS gene. It was shown that after transplantation of these cells to immunodeficient nude mice, the resulting epidermis was histologically equivalent to the epidermis of control animals, which were transplanted with healthy human keratinocytes. In addition, the barrier function of the transplanted mutant keratinocytes was close to the control [102].

To date, one clinical trial (NCT01545323) dedicated to the transplantation of skin graft genetically modified with a lentiviral vector encoding a functional copy of the serine peptidase inhibitor kazal type 5 (*SPINK5*) gene required for the formation of lympho-epithelial kazal-type-related inhibitor (LEKTI) protein in skin stem cells in Netherton Syndrome has been described. It has been shown that the normal shape and size of the upper layer of the skin was restored after genetic modification of defective keratinocytes in vitro [110].

Interestingly, a small number of cells carrying the wild-type *SPINK5* gene was sufficient to correct a large area of the graft. In the clinical trial, autologous epidermal layers made from genetically modified keratinocytes were used to treat patients with Netherton syndrome. The results of a phase I clinical trial have shown the practical applicability and safety of this approach. Genetically modified epithelial sheets were successfully formed and transplanted into three out of five patients (no suitable grafts could be obtained from the keratinocytes of the other two patients). The researchers published the results of one patient who was followed up for 12 months after successful transplantation. Recovery in the area of the graft was compared with the area outside. Morphology, the number of viral copies, and the level of the *SPINK5* gene expression were assessed. Successful engraftment of the graft, which completely merged with the surrounding skin, was observed. The number of viral copies could be determined only up to 3 months after transplantation (1–2 cycles of proliferation and differentiation of keratinocytes). There were no significant changes in TWEL levels before and after transplantation. The transplantation of genetically modified epidermal layers is considered a promising approach. The authors believe that the identification, targeting and engraftment of long-lived keratinocyte stem cell populations are required for a long-term therapeutic effect [103]. However, it is necessary to optimize this method to prolong the therapeutic effect.

For XLI therapy, Epstein–Barr virus (human herpesvirus type 4)-based vector encoding the STS gene was developed. It was shown that after genetic modification with this vector, the STS activity in the basal cells of the skin of an XLI patient was increased by about 100 times compared to normal keratinocytes. The researchers point out that the restoration of enzymatic activity led to the normal maturation of modified cells in vitro [106].

The use of programmed nucleases and small interfering RNAs can also be considered as a non-viral method of gene therapy. It is known that in the case of dominant-negative mutations, it is useless to replace the mutant gene with a healthy one, which is successfully used in the therapy of recessive mutation-induced diseases. Gene knockout is the most effective form of gene editing for dominant-negative mutations. Gene knockout can be achieved using programmable nucleases that are capable of cleaving certain regions of the genome and inactivating mutant alleles. For example, transcription activator-like effector nucleases (TALEN) can specifically target and cleave the DNA sequence, which often results in a frame shift and eventually activates a nonsense-mediated mRNA pathway [107,111].

In in vitro model of keratinocytes of a patient with epidermolytic ichthyosis caused by the mutations in the *KRT1* or *KRT10* genes, and in in vivo xenograft NOD/SCID mice, it was shown that TALEN specifically affects the *KRT10* region and leads to the complete degradation of mRNA encoded from the mutant *KRT10* gene, restoration of intermediate filament stability and changes in cellular and histological phenotypes [107].

Allele-specific small interfering RNAs can also be used for the correction of dominant-negative mutations, which has been studied, for example, for keratitis-ichthyosis-deafness syndrome. This is a severe, incurable disease characterized by ocular and skin abnormalities and hearing loss, with serious complications such as infections and skin cancer. The disease is often caused by dominant-negative mutations in the gap junction protein beta 2 (*GJB2*) gene encoding gap junction protein connexin 26. The use of mutant-allele-specific siRNA on the patient’s keratinocytes resulted in a strong inhibition of the mutant allele of the *GJB2* gene without altering the expression of the wild-type allele in vitro, which was also confirmed in NOD/SCID mice with skin grafts in vivo [86].

## 7. Conclusions

At the moment, there is pressing clinical need to find a safe, long-term and effective treatment for ichthyosis [47]. Even conventional approaches to the treatment of genetic diseases (such as ERT) are not reflected in the publications and apparently, poorly investigated. This is probably due to the fact that most forms of ichthyosis do not threaten the patient’s safety and appear only as skin lesions. Therefore, the most common type of treatment is symptomatic therapy, aiming at moisturizing and restoring the barrier function of the epidermis. The development of genetic engineering tools allowed for the production of new, radical approaches for the treatment of monogenic genetic diseases that affect the cause of the development of the disease. The use of viral vectors makes it possible to ensure a high-level expression of the gene of interest in cells of various types, including epithelial cells. However, gene therapy for ARCIs is only at the early stages of the development. The use of adenoviruses and herpes simplex viruses have not been investigated even in in vivo models, not to mention clinical trials. At the same time, gene therapy is being developed for other types of ichthyosis, and is considered the safest approach, allowing for a stable expression of the transgene without insertion into the genome. However, the direct administration of viral vectors has a number of disadvantages, such as insertional mutagenesis, which is common with retroviruses, and the induction of an immune response leading to inflammation. The introduction of viruses also inevitably leads to the formation of neutralizing antibodies, which significantly reduce the efficiency of subsequent transductions with the same virus. Moreover, the therapeutic effect of adenovirus-based and herpes simplex virus-based treatment may be offset due to the already high prevalence of anti-viral immunity in the human population. These disadvantages of gene therapy can be avoided by switching to cell-based drugs [112]. However, unfortunately, cell therapy approaches for ARCIs are not being investigated at all. Cell therapy has been described for only one type of ichthyosis, keratitis ichthyosis deafness syndrome (KID), characterized by limbal stem cell deficiency [113]. In KID, the ocular surface is seriously affected, while the surgical treatment is unfortunately unsuccessful [114]. The use of ocular surface stem cell transplantation has been shown to stabilize the ocular surface in KID patient [115]. Despite certain advances in the development of viral therapies for ichthyosis, there is still a long way to before an effective method of treatment is created. Nevertheless, gene therapy is the most promising of the existing approaches, since other methods do not solve the problem for a long time. However, due to the fact that most of the developed methods do not overcome the in vitro stage, the prospects for the development of ARCI gene therapy are very uncertain. Researchers must pay more active attention to this problem to make it possible to achieve high-efficacy ichthyosis therapy in the future.

## Figures and Tables

**Table 1:** Genes and associated ARCI phenotypes.

Gene	ARCI Phenotype	OMIM	References
*ALOX12B*	NBCIE, self-improving collodion ichthyosis (rare form)	#242100	[4,5,10]
*ALOXE3*	NBCIE, self-improving collodion ichthyosis (rare form)	#606545	[10,24]
*NIPAL4*/*ichthyin*	NBCIE	#612281	[8,25]
*PNPLA1*	NBCIE	#615024	[26]
*CYP4F22*	NBCIE	#604777	[5]
*ABCA12* truncating mutations	HI	#242500	[6]
*ABCA12* missense mutation	LI	#601277	[7,27]
*TGM1*	LI, bathing suit ichthyosis (rare form), self-improving collodion ichthyosis (rare form)	#242300	[9,10]
*CERS3*	Variable ARCI phenotype	#615023	[28]
*SDR9C7*	Variable ARCI phenotype	#617574	[29]
*SULT2B1*	Variable ARCI phenotype	#617571	[30]
*LIPN*	Variable ARCI phenotype	#613943	[31]

**Table 2:** Effectiveness of the currently available treatment approaches for autosomal recessive congenital ichthyosis.

Therapeutic Agent	Model	Therapy Results	Investigation Stage	References
ERT				
Liposomes with encapsulated recombinant human TGase-1 (rhTGase-1)	Skin-humanized mouse model	Restoration of TGase-1 activity and cholesterol gaps disappearance at the ultrastructural level	In vivo	[84]
TGase-1-loaded thermoresponsive nanogels (tNGs)	Skin equivalents generated from the fibroblasts and keratinocytes of ARCI patients	Significant decreases in apparent permeabilities, indicating improved barrier function	In vivo	[85]
Symptomatic Therapy				
Acitretin	Seven patients with LI	Two types of response: some patients responded to gradually increasing doses of oral retinoid (≥ 35 mg/day), others, at low doses (10–25 mg/day), and experienced worsening of the skin at higher doses	Clinical trial, completed	[70]
Isotretinoin, etretinate or acitretin	Patients with HI	An increase in survival rate by 59% compared to untreated children	Clinical trial, completed	[71]
Retinoids in combination with liarozol	Eleven LI patients and twelve healthy patients	A significant decrease in the expression of mRNA of the *KRT2* and *TNF-α* genes and a tendency to an increase in the expression of mRNA of the *KRT4* and *CYP26A1* genes	Clinical trial, completed	[75]
N-acetylcysteine	NIH3T3 fibroblast cells from LI patients	Inhibition of keratinocyte proliferation	Case report	[81]
Secukinumab	Paediatric patient with ABCA12-deficiency-related erythrodermic ichthyosis (ARCI) and efficacy and safety of a 16-weeks use of secukinumab in adult patients with ichthyosis	After the 6-month therapy period, there was a 48% reduction in the Ichthyosis Area Severity Index score	Clinical trial, completed	[78] NCT03041038
Imsidolimab (anti-IL-36 receptor monoclonal antibody)	Recruiting of patients with LI	-	Clinical trial, recruting	NCT04697056
Allele-specific small interfering RNA	Keratinocytes of patient with keratitis-ichthyosis-deafness syndrome, mouse xenograft model	Inhibition of the *GJB2* gene mutant allele without altering the expression of the wild-type allele		[86]

**Table 3:** Currently available gene therapy for ichthyosis.

Therapeutic Agent	Model	Therapy Results	Investigation Stage	References
Retrovirus encoding the *TGM1* gene	Keratinocytes of LI patient	Restoration of TGase-1 expression and activity in vitro	In vitro	[99]
HSV-1, encoding the *TGM1* gene	Immunocompetent BALB/c mice	Significant increase in TGase-1 expression in the epidermis without toxicity after both single and multiple local administrations in vivo	In vivo	[100]
HSV-1, encoding the *TGM1* gene	Patients with LI	No data	Clinical trial, active, not recruiting	NCT04047732
AVET system with plasmid DNA encoding the *TGM1* gene	Keratinocytes and organotypic cultures	The transfection efficiency was approximately 28%, which is much better than the results of transfection mediated by other polycationic transfection reagents	In vitro	[101]
Plasmid (pCMV-tag4B), encoding the *ABCA12* gene	Keratinocytes of HI patient	Restoration of lamellar granule lipid secretion and cell phenotypic rescue	In vitro	[6]
Keratinocytes of XLI patients transduced with retrovirus encoding the *STS* gene	Immunodeficient nude mice	An increase in the *STS* gene expression in vivo, normalization of the histological appearance 5 weeks after grafting, restoration of the barrier function	In vivo	[102]
Autologous epidermal sheets produced from genetically modified keratinocytes	Patients with Netherton syndrome	Complete engraftment, transgene expression duration up to 3 months, no significant changes in the levels of transepidermal water loss before and after transplantation	Clinical trial, active, not recruiting	NCT01545323 [103]
AAV2, encoding the *ALDH3A2* gene	Keratinocytes of patients with Sjögren–Larsson syndrome	Restoration of ALDH3A2 protein function, resistance to aldehydes increase almost to the normal level	In vitro	[104,105]
Epstein–Barr virus encoding the *STS* gene	Keratinocytes of XLI patients	An increase in STS activity by about 100 times, restoration of enzymatic activity led to the normal maturation of modified cells in vitro	In vitro	[106]
KRT10-specific TALEN	Keratinocytes of the patient with epidermolytic ichthyosis, mouse xenograft model	Complete degradation of mRNA of the mutant *KRT10* gene, restoration of intermediate filament stability and changes in cellular and histological phenotypes	In vivo	[107]
Allele-specific small interfering RNA	Keratinocytes of patient with keratitis-ichthyosis-deafness syndrome, mouse xenograft model	Inhibition of the *GJB2* gene mutant allele without altering the expression of the wild-type allele	In vivo	[86]

## References

[1] DiGiovanna J.J., Robinson-Bostom L. (2003). Ichthyosis: Etiology, diagnosis, and management. Am. J. Clin. Dermatol..

[2] Patel N., Spencer L.A., English J.C., Zirwas M.J. (2006). Acquired ichthyosis. J. Am. Acad. Dermatol..

[3] Oji V., Tadini G., Akiyama M., Blanchet Bardon C., Bodemer C., Bourrat E., Coudiere P., DiGiovanna J.J., Elias P., Fischer J. (2010). Revised nomenclature and classification of inherited ichthyoses: Results of the First Ichthyosis Consensus Conference in Soreze 2009. J. Am. Acad. Derm..

[4] Jobard F., Lefevre C., Karaduman A., Blanchet-Bardon C., Emre S., Weissenbach J., Ozguc M., Lathrop M., Prud’homme J.F., Fischer J. (2002). Lipoxygenase-3 (ALOXE3) and 12(R)-lipoxygenase (ALOX12B) are mutated in non-bullous congenital ichthyosiform erythroderma (NCIE) linked to chromosome 17p13.1. Hum. Mol. Genet..

[5] Lefevre C., Bouadjar B., Ferrand V., Tadini G., Megarbane A., Lathrop M., Prud’homme J.F., Fischer J. (2006). Mutations in a new cytochrome P450 gene in lamellar ichthyosis type 3. Hum. Mol. Genet..

[6] Akiyama M., Sugiyama-Nakagiri Y., Sakai K., McMillan J.R., Goto M., Arita K., Tsuji-Abe Y., Tabata N., Matsuoka K., Sasaki R. (2005). Mutations in lipid transporter ABCA12 in harlequin ichthyosis and functional recovery by corrective gene transfer. J. Clin. Investig..

[7] Kelsell D.P., Norgett E.E., Unsworth H., Teh M.T., Cullup T., Mein C.A., Dopping-Hepenstal P.J., Dale B.A., Tadini G., Fleckman P. (2005). Mutations in ABCA12 underlie the severe congenital skin disease harlequin ichthyosis. Am. J. Hum. Genet..

[8] Dahlqvist J., Klar J., Hausser I., Anton-Lamprecht I., Pigg M.H., Gedde-Dahl T., Ganemo A., Vahlquist A., Dahl N. (2007). Congenital ichthyosis: Mutations in ichthyin are associated with specific structural abnormalities in the granular layer of epidermis. J. Med. Genet..

[9] Oji V., Hautier J.M., Ahvazi B., Hausser I., Aufenvenne K., Walker T., Seller N., Steijlen P.M., Kuster W., Hovnanian A. (2006). Bathing suit ichthyosis is caused by transglutaminase-1 deficiency: Evidence for a temperature-sensitive phenotype. Hum. Mol. Genet..

[10] Vahlquist A., Bygum A., Ganemo A., Virtanen M., Hellstrom-Pigg M., Strauss G., Brandrup F., Fischer J. (2010). Genotypic and clinical spectrum of self-improving collodion ichthyosis: ALOX12B, ALOXE3, and TGM1 mutations in Scandinavian patients. J. Investig. Dermatol..

[11] Pigg M.H., Bygum A., Ganemo A., Virtanen M., Brandrup F., Zimmer A.D., Hotz A., Vahlquist A., Fischer J. (2016). Spectrum of Autosomal Recessive Congenital Ichthyosis in Scandinavia: Clinical Characteristics and Novel and Recurrent Mutations in 132 Patients. Acta Derm. Venereol..

[12] Rodriguez-Pazos L., Ginarte M., Fachal L., Toribio J., Carracedo A., Vega A. (2011). Analysis of TGM1, ALOX12B, ALOXE3, NIPAL4 and CYP4F22 in autosomal recessive congenital ichthyosis from Galicia (NW Spain): Evidence of founder effects. Br. J. Derm..

[13] Farasat S., Wei M.H., Herman M., Liewehr D.J., Steinberg S.M., Bale S.J., Fleckman P., Toro J.R. (2009). Novel transglutaminase-1 mutations and genotype-phenotype investigations of 104 patients with autosomal recessive congenital ichthyosis in the USA. J. Med. Genet..

[14] Herman M.L., Farasat S., Steinbach P.J., Wei M.H., Toure O., Fleckman P., Blake P., Bale S.J., Toro J.R. (2009). Transglutaminase-1 gene mutations in autosomal recessive congenital ichthyosis: Summary of mutations (including 23 novel) and modeling of TGase-1. Hum. Mutat.

[15] Pigg M., Gedde-Dahl T., Cox D., Hausser I., Anton-Lamprecht I., Dahl N. (1998). Strong founder effect for a transglutaminase 1 gene mutation in lamellar ichthyosis and congenital ichthyosiform erythroderma from Norway. Eur. J. Hum. Genet. EJHG.

[16] Esperon-Moldes U.S., Pardo-Seco J., Montalvan-Suarez M., Fachal L., Ginarte M., Rodriguez-Pazos L., Gomez-Carballa A., Moscoso F., Ugalde-Noritz N., Ordonez-Ugalde A. (2019). Biogeographical origin and timing of the founder ichthyosis TGM1 c.1187G > A mutation in an isolated Ecuadorian population. Sci. Rep..

[17] Eckert R.L., Kaartinen M.T., Nurminskaya M., Belkin A.M., Colak G., Johnson G.V., Mehta K. (2014). Transglutaminase regulation of cell function. Physiol. Rev..

[18] Akiyama M. (2011). Updated molecular genetics and pathogenesis of ichthiyoses. Nagoya J. Med. Sci..

[19] Akiyama M., Takizawa Y., Suzuki Y., Shimizu H. (2003). A novel homozygous mutation 371delA in TGM1 leads to a classic lamellar ichthyosis phenotype. Br. J. Derm..

[20] Akiyama M., Takizawa Y., Suzuki Y., Ishiko A., Matsuo I., Shimizu H. (2001). Compound heterozygous TGM1 mutations including a novel missense mutation L204Q in a mild form of lamellar ichthyosis. J. Investig. Dermatol..

[21] Akiyama M., Takizawa Y., Kokaji T., Shimizu H. (2001). Novel mutations of TGM1 in a child with congenital ichthyosiform erythroderma. Br. J. Derm..

[22] Fischer J. (2009). Autosomal recessive congenital ichthyosis. J. Investig. Dermatol..

[23] Simpson J.K., Martinez-Queipo M., Onoufriadis A., Tso S., Glass E., Liu L., Higashino T., Scott W., Tierney C., Simpson M.A. (2020). Genotype-phenotype correlation in a large English cohort of patients with autosomal recessive ichthyosis. Br. J. Derm..

[24] Wang T., Xu C., Zhou X., Li C., Zhang H., Lian B.Q., Lee J.J., Shen J., Liu Y., Lian C.G. (2015). Homozygous ALOXE3 Nonsense Variant Identified in a Patient with Non-Bullous Congenital Ichthyosiform Erythroderma Complicated by Superimposed Bullous Majocchi’s Granuloma: The Consequences of Skin Barrier Dysfunction. Int. J. Mol. Sci..

[25] Akbar A., Bint E.F.M., Crosby A.H., Gul A., Harlalka G.V. (2020). Variants in NIPAL4 and ALOXE3 cause autosomal recessive congenital ichthyosis in Pakistani families. Congenit. Anom..

[26] Zimmer A.D., Kim G.J., Hotz A., Bourrat E., Hausser I., Has C., Oji V., Stieler K., Vahlquist A., Kunde V. (2017). Sixteen novel mutations in PNPLA1 in patients with autosomal recessive congenital ichthyosis reveal the importance of an extended patatin domain in PNPLA1 that is essential for proper human skin barrier function. Br. J. Derm..

[27] Lefévre C., Audebert S., Jobard F., Bouadjar B., Lakhdar H., Boughdene-Stambouli O., Blanchet-Bardon C., Heilig R., Foglio M., Weissenbach J. (2003). Mutations in the transporter ABCA12 are associated with lamellar ichthyosis type 2. Hum. Mol. Genet..

[28] Radner F.P., Marrakchi S., Kirchmeier P., Kim G.J., Ribierre F., Kamoun B., Abid L., Leipoldt M., Turki H., Schempp W. (2013). Mutations in CERS3 cause autosomal recessive congenital ichthyosis in humans. PLoS Genet..

[29] Shigehara Y., Okuda S., Nemer G., Chedraoui A., Hayashi R., Bitar F., Nakai H., Abbas O., Daou L., Abe R. (2016). Mutations in SDR9C7 gene encoding an enzyme for vitamin A metabolism underlie autosomal recessive congenital ichthyosis. Hum. Mol. Genet..

[30] Heinz L., Kim G.J., Marrakchi S., Christiansen J., Turki H., Rauschendorf M.A., Lathrop M., Hausser I., Zimmer A.D., Fischer J. (2017). Mutations in SULT2B1 Cause Autosomal-Recessive Congenital Ichthyosis in Humans. Am. J. Hum. Genet..

[31] Israeli S., Khamaysi Z., Fuchs-Telem D., Nousbeck J., Bergman R., Sarig O., Sprecher E. (2011). A mutation in LIPN, encoding epidermal lipase N, causes a late-onset form of autosomal-recessive congenital ichthyosis. Am. J. Hum. Genet..

[32] Vahlquist A., Fischer J., Torma H. (2018). Inherited Nonsyndromic Ichthyoses: An Update on Pathophysiology, Diagnosis and Treatment. Am. J. Clin. Derm..

[33] Cottle D.L., Ursino G.M., Ip S.C., Jones L.K., Ditommaso T., Hacking D.F., Mangan N.E., Mellett N.A., Henley K.J., Sviridov D. (2015). Fetal inhibition of inflammation improves disease phenotypes in harlequin ichthyosis. Hum. Mol. Genet..

[34] Akiyama M., Kim D.K., Main D.M., Otto C.E., Holbrook K.A. (1994). Characteristic morphologic abnormality of harlequin ichthyosis detected in amniotic fluid cells. J. Investig. Dermatol..

[35] Akiyama M., Sakai K., Sato T., McMillan J.R., Goto M., Sawamura D., Shimizu H. (2007). Compound heterozygous ABCA12 mutations including a novel nonsense mutation underlie harlequin ichthyosis. Dermatology.

[36] Zhou X.J., Lin Y.J., Chen X.W., Zheng J.H., Zhou Y.J. (2021). Prenatal diagnosis of harlequin ichthyosis by ultrasonography: A case report. Ann. Transl. Med..

[37] Ganemo A., Pigg M., Virtanen M., Kukk T., Raudsepp H., Rossman-Ringdahl I., Westermark P., Niemi K.M., Dahl N., Vahlquist A. (2003). Autosomal recessive congenital ichthyosis in Sweden and Estonia: Clinical, genetic and ultrastructural findings in eighty-three patients. Acta Derm.-Venereol..

[38] Laiho E., Niemi K.M., Ignatius J., Kere J., Palotie A., Saarialho-Kere U. (1999). Clinical and morphological correlations for transglutaminase 1 gene mutations in autosomal recessive congenital ichthyosis. Eur. J. Hum. Genet. EJHG.

[39] Richard G., Adam M.P., Ardinger H.H., Pagon R.A., Wallace S.E., Bean L.J.H., Mirzaa G., Amemiya A. (1993). Autosomal Recessive Congenital Ichthyosis.

[40] Hohl D., Aeschlimann D., Huber M. (1998). In vitro and rapid in situ transglutaminase assays for congenital ichthyoses--a comparative study. J. Investig. Dermatol..

[41] Huber M., Rettler I., Bernasconi K., Frenk E., Lavrijsen S.P., Ponec M., Bon A., Lautenschlager S., Schorderet D.F., Hohl D. (1995). Mutations of keratinocyte transglutaminase in lamellar ichthyosis. Science.

[42] Akiyama M., Sawamura D., Shimizu H. (2003). The clinical spectrum of nonbullous congenital ichthyosiform erythroderma and lamellar ichthyosis. Clin. Exp. Dermatol..

[43] Yang C.S., Pomerantz H., Mannava K.A., Corwin J., Weinstock M.A., Fleckman P., DiGiovanna J.J., Robinson-Bostom L. (2016). Comparing histopathology from patients with X-linked recessive ichthyosis and autosomal recessive congenital ichthyosis with transglutaminase 1 mutation: A report from the National Registry for Ichthyosis and Related Skin Disorders. J. Am. Acad. Dermatol..

[44] Akiyama M. (1998). Severe congenital ichthyosis of the neonate. Int. J. Dermatol..

[45] Cheng R., Liang J., Li Y., Zhang J., Ni C., Yu H., Kong X., Li M., Yao Z. (2020). Next-generation sequencing through multi-gene panel testing for diagnosis of hereditary ichthyosis in Chinese. Clin Genet.

[46] Fioretti T., Auricchio L., Piccirillo A., Vitiello G., Ambrosio A., Cattaneo F., Ammendola R., Esposito G. (2020). Multi-Gene Next-Generation Sequencing for Molecular Diagnosis of Autosomal Recessive Congenital Ichthyosis: A Genotype-Phenotype Study of Four Italian Patients. Diagnostics.

[47] Paller A.S., Renert-Yuval Y., Suprun M., Esaki H., Oliva M., Huynh T.N., Ungar B., Kunjravia N., Friedland R., Peng X. (2017). An IL-17-dominant immune profile is shared across the major orphan forms of ichthyosis. J. Allergy Clin. Immunol..

[48] Malik K., He H., Huynh T.N., Tran G., Mueller K., Doytcheva K., Renert-Yuval Y., Czarnowicki T., Magidi S., Chou M. (2019). Ichthyosis molecular fingerprinting shows profound TH17 skewing and a unique barrier genomic signature. J. Allergy Clin. Immunol..

[49] Murase Y., Takeichi T., Kawamoto A., Tanahashi K., Okuno Y., Takama H., Shimizu E., Ishikawa J., Ogi T., Akiyama M. (2020). Reduced stratum corneum acylceramides in autosomal recessive congenital ichthyosis with a NIPAL4 mutation. J. Dermatol. Sci..

[50] O’Shaughnessy R.F., Choudhary I., Harper J.I. (2010). Interleukin-1 alpha blockade prevents hyperkeratosis in an in vitro model of lamellar ichthyosis. Hum. Mol. Genet..

[51] Matsuki M., Yamashita F., Ishida-Yamamoto A., Yamada K., Kinoshita C., Fushiki S., Ueda E., Morishima Y., Tabata K., Yasuno H. (1998). Defective stratum corneum and early neonatal death in mice lacking the gene for transglutaminase 1 (keratinocyte transglutaminase). Proc. Natl. Acad. Sci. USA.

[52] Candi E., Schmidt R., Melino G. (2005). The cornified envelope: A model of cell death in the skin. Nat. Rev. Mol. Cell Biol..

[53] Kuramoto N., Takizawa T., Takizawa T., Matsuki M., Morioka H., Robinson J.M., Yamanishi K. (2002). Development of ichthyosiform skin compensates for defective permeability barrier function in mice lacking transglutaminase 1. J. Clin. Investig..

[54] Garcia M., Larcher F., Hickerson R.P., Baselga E., Leachman S.A., Kaspar R.L., Del Rio M. (2011). Development of skin-humanized mouse models of pachyonychia congenita. J. Investig. Dermatol..

[55] Aufenvenne K., Rice R.H., Hausser I., Oji V., Hennies H.C., Rio M.D., Traupe H., Larcher F. (2012). Long-term faithful recapitulation of transglutaminase 1-deficient lamellar ichthyosis in a skin-humanized mouse model, and insights from proteomic studies. J. Investig. Dermatol..

[56] Briggaman R.A., Wheeler C.E. (1976). Lamellar ichthyosis: Long-term graft studies on congenitally athymic nude mice. J. Investig. Dermatol..

[57] Choate K.A., Khavari P.A. (1997). Direct cutaneous gene delivery in a human genetic skin disease. Hum. Gene. Ther..

[58] Mildner M., Ballaun C., Stichenwirth M., Bauer R., Gmeiner R., Buchberger M., Mlitz V., Tschachler E. (2006). Gene silencing in a human organotypic skin model. Biochem. Biophys. Res. Commun..

[59] Menon G.K. (2002). New insights into skin structure: Scratching the surface. Adv. Drug Deliv. Rev..

[60] Czarnowicki T., He H., Leonard A., Malik K., Magidi S., Rangel S., Patel K., Ramsey K., Murphrey M., Song T. (2018). The Major Orphan Forms of Ichthyosis Are Characterized by Systemic T-Cell Activation and Th-17/Tc-17/Th-22/Tc-22 Polarization in Blood. J. Investig. Dermatol..

[61] Randolph R.K., Simon M. (1993). Characterization of retinol metabolism in cultured human epidermal keratinocytes. J. Biol. Chem..

[62] Torma H., Rollman O., Vahlquist A. (1999). The vitamin A metabolism and expression of retinoid-binding proteins differ in HaCaT cells and normal human keratinocytes. Arch. Dermatol. Res..

[63] Karlsson T., Rollman O., Vahlquist A., Torma H. (2004). Immunofluorescence localization of nuclear retinoid receptors in psoriasis versus normal human skin. Acta Derm. -Venereol..

[64] Reichrath J., Mittmann M., Kamradt J., Muller S.M. (1997). Expression of retinoid-X receptors (-alpha,-beta,-gamma) and retinoic acid receptors (-alpha,-beta,-gamma) in normal human skin: An immunohistological evaluation. Histochem. J..

[65] Bastien J., Rochette-Egly C. (2004). Nuclear retinoid receptors and the transcription of retinoid-target genes. Gene.

[66] Eckert R.L., Welter J.F. (1996). Transcription factor regulation of epidermal keratinocyte gene expression. Mol. Biol. Rep..

[67] Brown L.J., Geesin J.C., Rothnagel J.A., Roop D.R., Gordon J.S. (1994). Retinoic acid suppression of loricrin expression in reconstituted human skin cultured at the liquid-air interface. J. Investig. Dermatol..

[68] Griffiths C.E., Rosenthal D.S., Reddy A.P., Elder J.T., Astrom A., Leach K., Wang T.S., Finkel L.J., Yuspa S.H., Voorhees J.J. (1992). Short-term retinoic acid treatment increases in vivo, but decreases in vitro, epidermal transglutaminase-K enzyme activity and immunoreactivity. J. Investig. Dermatol..

[69] Rosenthal D.S., Griffiths C.E., Yuspa S.H., Roop D.R., Voorhees J.J. (1992). Acute or chronic topical retinoic acid treatment of human skin in vivo alters the expression of epidermal transglutaminase, loricrin, involucrin, filaggrin, and keratins 6 and 13 but not keratins 1, 10, and 14. J. Investig. Dermatol..

[70] Khalil S., Bardawil T., Stephan C., Darwiche N., Abbas O., Kibbi A.G., Nemer G., Kurban M. (2017). Retinoids: A journey from the molecular structures and mechanisms of action to clinical uses in dermatology and adverse effects. J. Dermatol. Treat..

[71] Digiovanna J.J., Mauro T., Milstone L.M., Schmuth M., Toro J.R. (2013). Systemic retinoids in the management of ichthyoses and related skin types. Dermatol. Ther..

[72] Van Steensel M.A. (2007). Emerging drugs for ichthyosis. Expert Opin. Emerg. Drugs.

[73] Bryson H.M., Wagstaff A.J. (1996). Liarozole. Drugs Aging.

[74] Kang S., Duell E.A., Kim K.J., Voorhees J.J. (1996). Liarozole inhibits human epidermal retinoic acid 4-hydroxylase activity and differentially augments human skin responses to retinoic acid and retinol in vivo. J. Investig. Dermatol..

[75] Pavez Lorie E., Ganemo A., Borgers M., Wouters L., Blockhuys S., van de Plassche L., Torma H., Vahlquist A. (2009). Expression of retinoid-regulated genes in lamellar ichthyosis vs. healthy control epidermis: Changes after oral treatment with liarozole. Acta Derm. -Venereol..

[76] Gatzka M., Scharffetter-Kochanek K. (2015). T-cell plasticity in inflammatory skin diseases--the good, the bad, and the chameleons. J. Dtsch. Derm. Ges.

[77] Yamasaki K., Nakagawa H., Kubo Y., Ootaki K., Japanese Brodalumab Study G. (2017). Efficacy and safety of brodalumab in patients with generalized pustular psoriasis and psoriatic erythroderma: Results from a 52-week, open-label study. Br. J. Derm..

[78] Yogarajah J., Gouveia C., Iype J., Häfliger S., Schaller A., Nuoffer J., Fux M., Gautschi M. (2021). Efficacy and safety of secukinumab for the treatment of severe ABCA12 deficiency-related ichthyosis in a child. Ski. Health Dis..

[79] Oji V., Traupe H. (2009). Ichthyosis: Clinical manifestations and practical treatment options. Am. J. Clin. Dermatol..

[80] Tadini G., Giustini S., Milani M. (2011). Efficacy of topical 10% urea-based lotion in patients with ichthyosis vulgaris: A two-center, randomized, controlled, single-blind, right-vs.-left study in comparison with standard glycerol-based emollient cream. Curr. Med. Res. Opin..

[81] Bassotti A., Moreno S., Criado E. (2011). Successful treatment with topical N-acetylcysteine in urea in five children with congenital lamellar ichthyosis. Pediatr. Dermatol..

[82] Allen A., Siegfried E., Silverman R., Williams M.L., Elias P.M., Szabo S.K., Korman N.J. (2001). Significant absorption of topical tacrolimus in 3 patients with Netherton syndrome. Arch. Dermatol..

[83] Halverstam C.P., Vachharajani A., Mallory S.B. (2007). Cushing syndrome from percutaneous absorption of 1% hydrocortisone ointment in Netherton syndrome. Pediatr. Dermatol..

[84] Aufenvenne K., Larcher F., Hausser I., Duarte B., Oji V., Nikolenko H., Del Rio M., Dathe M., Traupe H. (2013). Topical enzyme-replacement therapy restores transglutaminase 1 activity and corrects architecture of transglutaminase-1-deficient skin grafts. Am. J. Hum. Genet..

[85] Plank R., Yealland G., Miceli E., Lima Cunha D., Graff P., Thomforde S., Gruber R., Moosbrugger-Martinz V., Eckl K., Calderon M. (2019). Transglutaminase 1 Replacement Therapy Successfully Mitigates the Autosomal Recessive Congenital Ichthyosis Phenotype in Full-Thickness Skin Disease Equivalents. J. Investig. Dermatol..

[86] Lee M.Y., Wang H.Z., White T.W., Brooks T., Pittman A., Halai H., Petrova A., Xu D., Hart S.L., Kinsler V.A. (2020). Allele-Specific Small Interfering RNA Corrects Aberrant Cellular Phenotype in Keratitis-Ichthyosis-Deafness Syndrome Keratinocytes. J. Investig. Dermatol..

[87] Uthoff D., Gorney M., Teichmann C. (1994). Cicatricial ectropion in ichthyosis: A novel approach to treatment. Ophthalmic. Plast. Reconstr. Surg..

[88] Das S., Honavar S.G., Dhepe N., Naik M.N. (2010). Maternal skin allograft for cicatricial ectropion in congenital icthyosis. Ophthalmic. Plast. Reconstr. Surg..

[89] Rybarova N., Pinkova B., Doskova H., Vlkova E. (2020). Sight-threatening Complication of Cicatricial Ectropion in a Patient with Lamellar Ichthyosis—Case Report. Acta Dermatovenerol. Croat. ADC.

[90] Subramanian N., Nivean P.D., Alam M.S. (2020). Combined medical and surgical management for cicatricial ectropion in lamellar ichthyosis: A report of three cases. Indian J. Ophthalmol..

[91] Schaefer R.M., Tylki-Szymanska A., Hilz M.J. (2009). Enzyme replacement therapy for Fabry disease: A systematic review of available evidence. Drugs.

[92] Okuyama T., Tanaka A., Suzuki Y., Ida H., Tanaka T., Cox G.F., Eto Y., Orii T. (2010). Japan Elaprase Treatment (JET) study: Idursulfase enzyme replacement therapy in adult patients with attenuated Hunter syndrome (Mucopolysaccharidosis II, MPS II). Mol. Genet. Metab..

[93] Tanaka N., Saito H., Ito T., Momose K., Ishida F., Hora K., Kiyosawa K., Ida H. (2001). Initiation of enzyme replacement therapy for an adult patient with asymptomatic type 1 Gaucher’s disease. Intern. Med..

[94] Solovyeva V.V., Shaimardanova A.A., Chulpanova D.S., Kitaeva K.V., Chakrabarti L., Rizvanov A.A. (2018). New Approaches to Tay-Sachs Disease Therapy. Front. Physiol..

[95] Shaimardanova A.A., Chulpanova D.S., Solovyeva V.V., Mullagulova A.I., Kitaeva K.V., Allegrucci C., Rizvanov A.A. (2020). Metachromatic Leukodystrophy: Diagnosis, Modeling, and Treatment Approaches. Front. Med..

[96] Cuggino J.A., Alvarez C.I.I., Strumia M.C., Welker P., Licha K., Steinhilber D., Mutihac R.-C., Calderon M. (2011). Thermosensitive nanogels based on dendritic polyglycerol and N-isopropylacrylamide for biomedical applications. Soft Matter..

[97] Witting M., Molina M., Obst K., Plank R., Eckl K.M., Hennies H.C., Calderon M., Friess W., Hedtrich S. (2015). Thermosensitive dendritic polyglycerol-based nanogels for cutaneous delivery of biomacromolecules. Nanomedicine.

[98] Siprashvili Z., Nguyen N.T., Bezchinsky M.Y., Marinkovich M.P., Lane A.T., Khavari P.A. (2010). Long-term type VII collagen restoration to human epidermolysis bullosa skin tissue. Hum. Gene Ther..

[99] Choate K.A., Kinsella T.M., Williams M.L., Nolan G.P., Khavari P.A. (1996). Transglutaminase 1 delivery to lamellar ichthyosis keratinocytes. Hum. Gene Ther..

[100] Freedman J.C., Parry T.J., Zhang P., Majumdar A., Krishnan S., Regula L.K., O’Malley M., Coghlan S., Yogesha S.D., Ramasamy S. (2021). Preclinical Evaluation of a Modified Herpes Simplex Virus Type 1 Vector Encoding Human TGM1 for the Treatment of Autosomal Recessive Congenital Ichthyosis. J. Investig. Dermatol..

[101] Huber M., Limat A., Wagner E., Hohl D. (2000). Efficient in vitro transfection of human keratinocytes with an adenovirus-enhanced receptor-mediated system. J. Investig. Dermatol..

[102] Freiberg R.A., Choate K.A., Deng H., Alperin E.S., Shapiro L.J., Khavari P.A. (1997). A model of corrective gene transfer in X-linked ichthyosis. Hum. Mol. Genet..

[103] Di W.L., Lwin S.M., Petrova A., Bernadis C., Syed F., Farzaneh F., Moulding D., Martinez A.E., Sebire N.J., Rampling D. (2019). Generation and Clinical Application of Gene-Modified Autologous Epidermal Sheets in Netherton Syndrome: Lessons Learned from a Phase 1 Trial. Hum. Gene Ther..

[104] Haug S., Braun-Falco M. (2005). Adeno-associated virus vectors are able to restore fatty aldehyde dehydrogenase-deficiency. Implications for gene therapy in Sjogren-Larsson syndrome. Arch. Dermatol. Res..

[105] Haug S., Braun-Falco M. (2006). Restoration of fatty aldehyde dehydrogenase deficiency in Sjogren-Larsson syndrome. Gene. Ther..

[106] Jensen T.G., Jensen U.B., Jensen P.K., Ibsen H.H., Brandrup F., Ballabio A., Bolund L. (1993). Correction of steroid sulfatase deficiency by gene transfer into basal cells of tissue-cultured epidermis from patients with recessive X-linked ichthyosis. Exp. Cell Res..

[107] March O.P., Lettner T., Klausegger A., Ablinger M., Kocher T., Hainzl S., Peking P., Lackner N., Rajan N., Hofbauer J.P. (2019). Gene Editing-Mediated Disruption of Epidermolytic Ichthyosis-Associated KRT10 Alleles Restores Filament Stability in Keratinocytes. J. Investig. Dermatol..

[108] Gorell E., Nguyen N., Lane A., Siprashvili Z. (2014). Gene therapy for skin diseases. Cold Spring Harb. Perspect. Med..

[109] Naso M.F., Tomkowicz B., Perry W.L., Strohl W.R. (2017). Adeno-Associated Virus (AAV) as a Vector for Gene Therapy. BioDrugs Clin. Immunother. Biopharm. Gene Ther..

[110] Di W.L., Mellerio J.E., Bernadis C., Harper J., Abdul-Wahab A., Ghani S., Chan L., Martinez-Queipo M., Hara H., McNicol A.M. (2013). Phase I study protocol for ex vivo lentiviral gene therapy for the inherited skin disease, Netherton syndrome. Hum. Gene Ther. Clin. Dev..

[111] Nemudryi A.A., Valetdinova K.R., Medvedev S.P., Zakian S.M. (2014). TALEN and CRISPR/Cas Genome Editing Systems: Tools of Discovery. Acta Nat..

[112] Goswami R., Subramanian G., Silayeva L., Newkirk I., Doctor D., Chawla K., Chattopadhyay S., Chandra D., Chilukuri N., Betapudi V. (2019). Gene Therapy Leaves a Vicious Cycle. Front. Oncol..

[113] Caceres-Rios H., Tamayo-Sanchez L., Duran-Mckinster C., de la Luz Orozco M., Ruiz-Maldonado R. (1996). Keratitis, ichthyosis, and deafness (KID syndrome): Review of the literature and proposal of a new terminology. Pediatr. Derm..

[114] Maguire M.G., Stark W.J., Gottsch J.D., Stulting R.D., Sugar A., Fink N.E., Schwartz A. (1994). Risk factors for corneal graft failure and rejection in the collaborative corneal transplantation studies. Collaborative Corneal Transplantation Studies Research Group. Ophthalmology.

[115] Cheung A.Y., Patel S., Kurji K.H., Sarnicola E., Eslani M., Govil A., Holland E.J. (2019). Ocular Surface Stem Cell Transplantation for Treatment of Keratitis-Ichthyosis-Deafness Syndrome. Cornea.

